# A Randomized, Double-Blind, Placebo-Controlled Study of HLD200, a Delayed-Release and Extended-Release Methylphenidate, in Children with Attention-Deficit/Hyperactivity Disorder: An Evaluation of Safety and Efficacy Throughout the Day and Across Settings

**DOI:** 10.1089/cap.2019.0070

**Published:** 2020-01-30

**Authors:** Ann C. Childress, Andrew J. Cutler, Andrea Marraffino, Mary Ann McDonnell, John M. Turnbow, Matthew Brams, Norberto J. DeSousa, Bev Incledon, Floyd R. Sallee, Sharon B. Wigal

**Affiliations:** ^1^ Center for Psychiatry and Behavioral Medicine, Inc., Las Vegas, Nevada.; ^2^ Meridien Research, Bradenton, Florida.; ^3^ SUNY Upstate Medical University, Department of Psychiatry, Syracuse, New York.; ^4^ Meridien Research, Maitland, Florida.; ^5^ South Shore Psychiatric Services, Hingham, Massachusetts.; ^6^ Westex Clinical Investigations, Lubbock, Texas.; ^7^ Bayou City Research, Ltd., Houston, Texas.; ^8^ Ironshore Pharmaceuticals & Development, Inc., Camana Bay, Grand Cayman, Cayman Islands.; ^9^ Ironshore Pharmaceuticals Inc., Durham, North Carolina.; ^10^ AVIDA, Inc., Newport Beach, California.

**Keywords:** methylphenidate, attention-deficit/hyperactivity disorder, functional impairment, DR/ER-MPH, duration, safety

## Abstract

***Objectives:*** HLD200, a once-daily, evening-dosed, delayed-release and extended-release methylphenidate (DR/ER-MPH), was designed to provide therapeutic effect beginning upon awakening and lasting into the evening. This pivotal, randomized, double-blind, multicenter, placebo-controlled, phase 3 trial assessed improvements in functional impairment across the day using multiple validated measures tailored for different settings and time of day in children (6–12 years) with attention-deficit/hyperactivity disorder (ADHD).

***Methods:*** Following a 6-week, open-label titration of DR/ER-MPH to an optimal dose (20, 40, 60, 80, or 100 mg/day) and dosing time (8:00 PM ±1.5 hours), participants were randomized to treatment-optimized DR/ER-MPH or placebo for 1 week. The primary endpoint was the model-adjusted average of postdose Swanson, Kotkin, Agler, M-Flynn, and Pelham Scale combined scores (SKAMP CS) over a 12-hour laboratory classroom day (8:00 AM to 8:00 PM). The key secondary endpoint was the Parent Rating of Evening and Morning Behavior-Revised, Morning (PREMB-R AM) subscale. Secondary/exploratory measures included the PREMB-R Evening (PREMB-R PM) subscale and Permanent Product Measure of Performance (Attempted [PERMP-A] and Correct [PERMP-C]). Safety endpoints included treatment-emergent adverse events (TEAEs).

***Results:*** After the treatment-optimization phase, the mean optimized dose was 66.2 mg and the most common prescribed dosing time was 8:00 PM. Double-blind DR/ER-MPH treatment significantly improved functional impairment versus placebo in the early morning (PREMB-R AM: *p* < 0.001), averaged over the classroom day (SKAMP CS: *p* < 0.001), and in the late afternoon/evening (PREMB-R PM: *p* = 0.003) in the intent-to-treat population (*N* = 117). Average PERMP-A (*p* = 0.006) and PERMP-C (*p* = 0.009) also indicated improved classroom performance with DR/ER-MPH versus placebo. In the double-blind phase, TEAEs did not differ between DR/ER-MPH and placebo groups and no serious TEAEs or TEAEs leading to discontinuation were reported.

***Conclusion:*** DR/ER-MPH was well tolerated and demonstrated significant improvements versus placebo in functional impairment throughout the day across different settings in children with ADHD.

## Introduction

Patients with attention-deficit/hyperactivity disorder (ADHD) seek treatment primarily due to functional impairment (e.g., strained relations, academic underperformance, job loss, or lack of friends), while physicians have historically focused on achieving symptom control (Weiss et al. 2018). The Diagnostic and Statistical Manual of Mental Disorders, 5th ed. (DSM-5) mandates that symptoms interfere with functioning in two or more settings (American Psychiatric Association 2013); however, it does not offer guidance to physicians on how to operationalize functional impairment in a clinically relevant way (Ustün and Kennedy 2009). At the same time, current treatment guidelines underscore the significance of achieving normative functioning as a goal (Pliszka and AACAP Work Group on Quality Issues 2007; Subcommittee on Attention-Deficit/Hyperactivity Disorder et al. 2011). In school-aged children, settings in which functional impairment occur are generally confined to specific times of the day, for example, family interactions in the early morning; classroom activities and peer relations throughout the day; extracurricular activities, homework, and self-care in the late afternoon and evening.

While commonly prescribed long-acting methylphenidate (MPH) formulations, which are recommended as first-line treatment for ADHD (Pliszka and AACAP Work Group on Quality Issues 2007; Subcommittee on Attention-Deficit/Hyperactivity Disorder et al. 2011), are given once daily in the morning to control ADHD symptoms into the afternoon or evening depending on the delivery system (Childress 2016; Childress et al. 2017), they do not provide therapeutic coverage for all parts of the day. All long-acting MPH formulations have an inherent delay between dosing and onset of therapeutic effect, which results in inadequate control during the early morning (Sallee 2015; Faraone et al. 2017), a particularly challenging time of the day for school-age children with ADHD and their families (Whalen et al. 2006; Sallee 2015; Faraone et al. 2017). Therefore, there remains a significant unmet need in stimulant-treated youth with ADHD to provide clinically meaningful control of ADHD symptoms and/or functional impairment from the early morning and lasting into the evening (Sallee 2015; Faraone et al. 2017).

While the delay between medication administration and onset of therapeutic effect cannot be eliminated, it can be extended in a precise and controlled manner. HLD200 (trade name JORNAY PM^TM^) is a once-daily, evening-dosed, delayed-release and extended-release MPH (DR/ER-MPH) formulation. The pharmacokinetic profile of DR/ER-MPH is characterized by delay of MPH absorption until the early morning, with <5% of total MPH available within the first 10 hours after evening administration, followed by extended, controlled release throughout the day (Childress et al. 2018; Liu et al. 2019). DR/ER-MPH capsules include hundreds of identical microbeads that have two functional layers. Based on the properties of the outer DR and inner ER layers, dissolution and subsequent absorption of MPH occur independently of any single factor, such as pH trigger, normal variations in gastrointestinal transit, or site of release (Childress et al. 2018; Liu et al. 2019).

In the current study, the clinical benefits of DR/ER-MPH were assessed using validated rating scales that are tailored for different times of the day and reflect appropriate settings and raters for the early morning, the school day, and the evening. The primary objective of the current phase 3, randomized, placebo-controlled, double-blind study was to assess whether treatment-optimized, evening-dosed DR/ER-MPH improves ADHD-related functional impairment, compared with placebo, throughout the school-day period (8:00 AM to 8:00 PM) in children with ADHD. In addition, early morning and late afternoon/evening functional impairment as well as safety and tolerability were assessed.

## Methods

### Study conduct

This phase 3, double-blind, randomized, placebo-controlled, parallel group study of treatment-optimized DR/ER-MPH ending with a laboratory classroom study day in children 6–12 years of age with ADHD was conducted from March 2016 through July 2016 at seven sites in the United States. The study was conducted in accordance with the Declaration of Helsinki and Good Clinical Practice guidelines, and all participants and parents/legal guardians provided informed assent and consent, respectively, under procedures approved by each site's Institutional Review Board.

### Inclusion/exclusion criteria

Children (6–12 years) diagnosed with ADHD as defined by DSM-5 criteria (American Psychiatric Association 2013) and confirmed using the Mini International Neuropsychiatric Interview for Children and Adolescents (MINI-KID) (Sheehan et al. 2010) were enrolled if they met the defined study inclusion and exclusion criteria. Inclusion criteria included, but were not limited to: ADHD Rating Scale based on Diagnostic and Statistical Manual of Mental Disorders, 4th ed. (DSM-IV; American Psychiatric Association 1994) criteria (ADHD-RS-IV) score ≥90th percentile at baseline for age and sex in at least one of the following categories: hyperactive-impulsive, inattentive, or total score, and a total score of ≥26 at baseline (DuPaul et al. 1998); Clinical Global Impressions of Severity (CGI-S) score ≥4 (Guy 1976) and a Conners' Global Index-Parent (CGI-P) score >10 (Conners 1989) at baseline; participants who were not at the time on MPH treatment had to either have prior experience with MPH treatment showing clinical response to therapy during that time, or had to be treated with the same dose of MPH to which they had shown a clinical response with acceptable tolerability to MPH for ≥2 weeks before screening; parent/guardian confirmation of early morning functional (EMF) impairment by history and difficulties performing a morning routine; regular weekday morning routine of ≥30 minutes; and negative pregnancy test and a form of birth control for female participants of childbearing potential.

Exclusion criteria included, but were not limited to: history of or current medical condition or laboratory result that could interfere with study participation, participant safety, or satisfactory completion of the study; any cardiac problems that may have placed the participant at increased vulnerability to the sympathomimetic effects of a stimulant drug; history of psychosis, bipolar disorder, anorexia nervosa, bulimia, or suicide attempt; current depression, anxiety, conduct disorder, substance use disorder, or other psychiatric condition which may have jeopardized participant safety or interfered with the satisfactory completion of the study; history of severe allergic reaction or intolerance to MPH; positive screening for illicit drug use or nicotine use; and/or current health conditions or use of medications that may have increased risk to the participant or confounded the results of the study.

### Study design

**FIG. 1.**
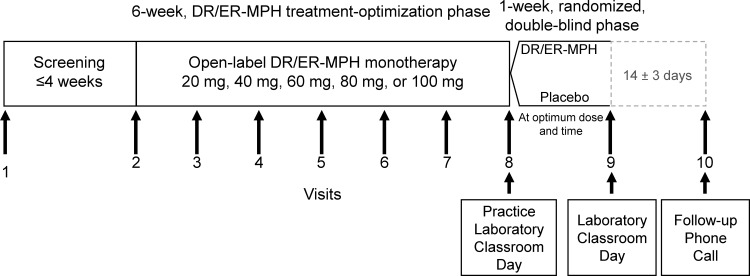
Study design. DR/ER-MPH, delayed-release and extended-release methylphenidate.

This trial was conducted in three distinct phases: a screening/washout phase of up to 4 weeks, a 6-week, open-label, DR/ER-MPH treatment-optimization phase, and a 1-week double-blind, randomized, placebo-controlled, parallel-group phase ending with a laboratory classroom study day (Fig. 1). During the screening period of up to 4 weeks (starting at Visit 1), participants meeting the entry criteria who were currently taking ADHD medication were withdrawn from these medication(s) for a minimum of 5 days before beginning the open-label, DR/ER-MPH treatment-optimization phase.

At the start of the 6-week, open-label, DR/ER-MPH treatment-optimization phase (baseline/Visit 2), baseline measurements of vital signs and appropriate scales were taken, and participants initiated once-daily evening (8:00 PM ±30 minutes) treatment with 20 or 40 mg DR/ER-MPH (based on prior treatment history) for 1 week. Up to four subsequent weekly dosage adjustments (Visits 3–6) were permitted to achieve both an optimal daily dose (20, 40, 60, 80, or 100 mg) and an optimal evening administration time (8:00 PM ±1.5 hours) before the start of the double-blind, placebo-controlled test phase. Dose titrations were permitted in 20- or 40-mg increments or decrements until an optimal daily dose was achieved or a maximum daily dose of 100 mg/day or 3.7 mg/kg (based on baseline body weight) was reached. Administration time adjustments were permitted in increments or decrements of 30–60 minutes until an optimal treatment time between 6:30 PM and 9:30 PM was achieved. The final permitted dose level or administration time adjustment was at Visit 6, after which, participants were maintained on their optimized dose and evening administration time from Visit 7 to randomization at Visit 8. Optimal daily dose and evening administration time were defined as those that produced meaningful control of symptoms throughout the day and EMF impairment while remaining safe and well tolerated with a minimum of ≥33% improvement in total score from baseline (Visit 2) to randomization (Visit 8) for ADHD-RS-IV, Before School Functioning Questionnaire (BSFQ), and CGI-P. In addition, participants had to demonstrate a stable BSFQ score as defined by a difference of <10 points between Visits 7 and 8.

The BSFQ is a validated, 20-item instrument evaluating EMF impairment in children with ADHD between the time of awakening and before the school day (Wilens et al. 2010; Faraone et al. 2018b). Clinicians rated items based on a structured parental survey; additionally, clinicians recorded the time to complete early morning routines, from getting out of bed to exiting the home.

At the start of the double-blind, placebo-controlled phase (Visit 8), participants were randomized (1:1) to receive either DR/ER-MPH (optimal dose) or placebo once-daily at the optimal evening administration time for 1 week. Participants returned to the clinic 1 week after randomization (Visit 9) for the laboratory school day (Swanson et al. 2000, 2002) consisting of nine classroom sessions and other assessments. For all assessments, each site was to make all efforts to maintain the same qualified rater for each individual participant across measurements.

### Efficacy endpoints

The primary efficacy endpoint was the model-adjusted average of all postdose Swanson, Kotkin, Agler, M-Flynn, and Pelham Scale combined scores (SKAMP CS) measured on the laboratory classroom day (Visit 9) during the 12-hour time period from 8:00 AM to 8:00 PM. The key secondary efficacy endpoint was the Parent Rating of Evening and Morning Behavior-Revised, Morning (PREMB-R AM) subscale and other secondary/exploratory endpoints included the PREMB-R Evening (PREMB-R PM) subscale, SKAMP CS at individual time points, and Permanent Product Measure of Performance (PERMP) average scores during the 12-hour classroom day and at individual time points.

Postdose SKAMP ratings were measured on Visit 9 at each of the nine laboratory classroom assessments occurring at 8:00 AM, 9:00 AM, 10:00 AM, 12:00 PM, 2:00 PM, 4:00 PM, 6:00 PM, 7:00 PM, and 8:00 PM (each ±15 minutes). The SKAMP rating scale is a validated 13-item scale of impairment based on observer ratings of classroom behaviors during laboratory school assessments (Swanson et al. 1998, 2000, 2002; Wigal et al. 1998; Wigal and Wigal 2006). Each item is rated from 0 (no impairment) to 6 (maximal impairment), with a maximum combined score of 78.

The 11-item PREMB-R is a validated scale that assesses functional impairment based on behaviors (e.g., getting up and out of bed, doing or completing homework, sitting through dinner, and getting to bed and falling asleep) during the early morning (PREMB-R AM subscale; three items) and late afternoon/evening periods (PREMB-R PM subscale; eight items) in children with ADHD (Sutton et al. 2003; Faraone et al. 2018a). Each item is rated from 0 (no difficulty) to 3 (a lot of difficulty), with the three-item PREMB-R AM having a maximum score of 9, and the eight-item PREMB-R PM having a maximum score of 24. PREMB-R results were investigator-rated summary scores derived from clinician-administered parent interviews, with the parent PREMB-R ratings based on the last 2 weekdays before the laboratory classroom day (Visit 9).

The PERMP is a validated 10-minute written mathematics test performed as seatwork in the laboratory school analog classroom (Swanson et al. 1998; Wigal and Wigal 2006). Performance is measured by the number of problems attempted (PERMP-A) and the number of problems correctly completed (PERMP-C). Appropriate difficulty levels of the mathematics tests were determined by the PERMP pretest conducted at Visit 2. In addition, different versions of the mathematics tests for a given difficulty level were used across class sessions, so that a participant did not take the same test more than once during the study. PERMP assessments were administered as practice tests at Visit 8 during three to four practice day classroom sessions and as double-blind tests at Visit 9 during each of nine classroom sessions.

### Safety

Safety endpoints included spontaneously reported treatment-emergent adverse events (TEAEs). TEAEs of special interest included appetite suppression and insomnia, with sleep disturbances (onset, quality, and quantity) directly queried from participants and parents at each visit, from informed consent through the follow-up call 14 ± 3 days after final dose. TEAEs were recorded in the phase in which they occurred or worsened (e.g., a TEAE that occurred in the open-label phase and continued without worsening into the double-blind phase was only counted in the open-label phase). Sleep-related TEAEs were summarized using the preferred terms “initial insomnia”, “middle insomnia”, “terminal insomnia”, and “insomnia (not specified)” based on the Version 18.0 of the Medical Dictionary for Regulatory Affairs and the combined rate of all sleep disturbances was reported as “any insomnia”. Other safety assessments included vital signs, electrocardiogram parameters, clinical laboratory test results, physical examination findings, and measurements from the Columbia-Suicide Severity Rating Scale for Children (Posner et al. 2011).

### Statistical analyses

The study planned to screen ∼150 participants to randomize at least 120 participants into the double-blind placebo-controlled test phase (Visit 8 through Visit 9). Analyses were conducted on the intent-to-treat (ITT) and safety populations. The enrolled safety population was defined as all enrolled participants who received at least one dose of study drug and had at least one postbaseline safety assessment; this population was used to conduct safety analyses for the open-label, treatment-optimization phase. The randomized safety population included participants who received at least one dose of double-blind study drug and had at least one postbaseline safety assessment; this population was used for comparative safety analyses between DR/ER-MPH and placebo groups for the double-blind phase. The ITT population, the basis for the primary and secondary efficacy analyses, was defined as all randomized participants who received at least one dose of double-blind study drug and had at least one postbaseline evaluation of the primary efficacy assessment.

**FIG. 2.**
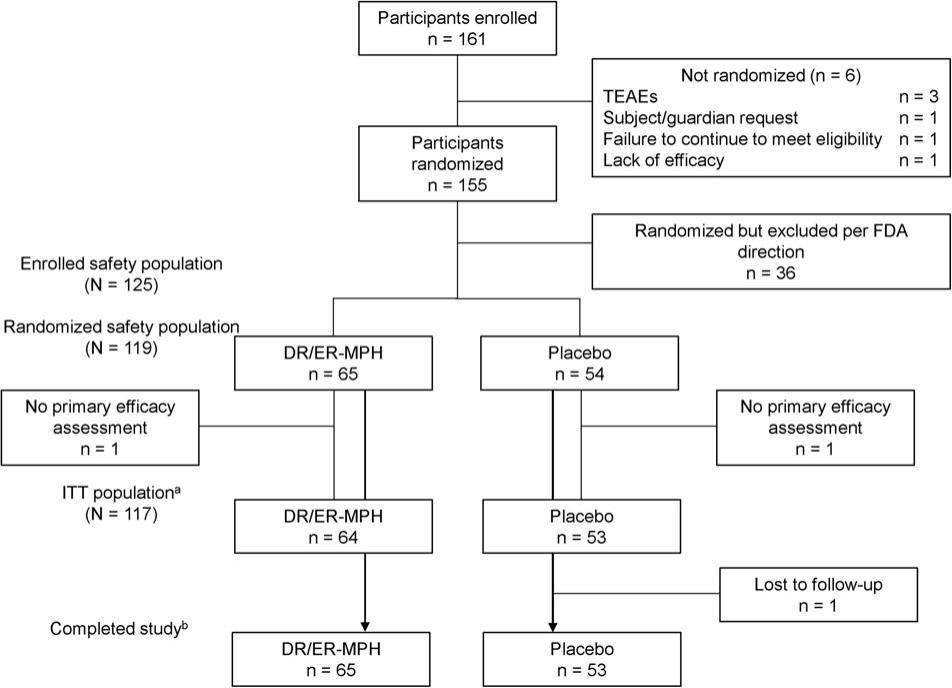
Participant disposition. ^a^The ITT population was defined as all randomized participants who received at least one dose of double-blind study drug and had at least one postbaseline evaluation of the primary efficacy assessment. ^b^Participants who completed the study were those who completed the follow-up phone call 14 ± 3 days following the laboratory classroom day. TEAE, treatment-emergent adverse event; FDA, United States Food and Drug Administration; DR/ER-MPH, delayed-release and extended-release methylphenidate; ITT, intent-to-treat.

Two of the study sites (referred to as Site A and B) warranted further examination. Following an independent audit, and their own inspection, the United States Food and Drug Administration removed Site A (*n* = 36) from both efficacy and safety analyses due to concerns about data integrity (United States Food and Drug Administration 2018). Following the site removal, the enrolled safety, randomized safety, and ITT populations included 125, 119, and 117 participants, respectively (Fig. 2).

During study conduct, it was discovered that up to 15 of the 17 participants enrolled in Cohort 2 at Site B were assigned multiple randomization numbers because of a shipping error that resulted in an inventory shortage of investigational product (United States Food and Drug Administration 2018). These participants were included in all ITT and safety analyses and were analyzed according to the treatment associated with the randomization number ultimately used. To investigate whether the randomization error affected treatment outcomes, sensitivity analyses of primary and key secondary endpoints were performed on a sensitivity population, which consisted of the ITT population, excluding the 17 participants in Cohort 2 of Site B.

SKAMP CS was analyzed using a mixed model repeated-measures (MMRM) analysis that included the following prespecified variables: participant's intercept as a random effect and treatment, study center, time point, and time point by treatment interaction as fixed effects. The average treatment difference over all postdose time points was estimated using least squares (LS) mean from the MMRM and standard errors (SEs) were calculated. Treatment comparisons were conducted as two-sided tests at the 5% level of significance. SKAMP CS at individual time points were analyzed based on the same MMRM used for the primary efficacy analysis. Because participants entered the laboratory classroom treated from dosing the prior evening, no baseline adjustment was performed for SKAMP CS measurements.

PREMB-R AM and PREMB-R PM at Visit 9 were assessed by using an analysis of covariance model with treatment as the main effect and study center and baseline score at baseline (Visit 2) as the covariates. PERMP efficacy variables were analyzed based on the same MMRM used for the primary efficacy analysis.

## Results

### Participant disposition, demographics, and baseline characteristics

The trial enrolled 161 participants. Following the removal of one site (*n* = 36) from both safety and efficacy analyses, the enrolled safety population consisted of 125 participants, the randomized safety population consisted of 119 participants (DR/ER-MPH, *n* = 65; placebo, *n* = 54), and the ITT population consisted of 117 participants (DR/ER-MPH, *n* = 64; placebo, *n* = 53) (Fig. 2). Of the 125 participants in the enrolled safety population, six discontinued the study before randomization (three because of a TEAE; one because of participant or parent/guardian request; one because of failure to continue to meet eligibility criteria, and one because of lack of efficacy). Two randomized participants (one in each of the placebo and DR/ER-MPH groups) were excluded from the ITT population because they failed to attend the assessments at Visit 9. One participant randomized to the placebo group completed Visit 9 assessments but discontinued from the study due to loss to follow-up.

The demographic and baseline characteristics of the enrolled and randomized safety populations are shown in Table 1. The demographic and baseline characteristics of the DR/ER-MPH and placebo groups were generally comparable with a few exceptions (Table 1). Compared with the placebo group, the DR/ER-MPH group had a slightly higher percentage of participants 11–12 years of age (32.3% vs. 25.9%) and participants categorized as severely ill on the CGI-S at baseline (24.6% vs. 9.3%). The DR/ER-MPH group also had a lower percentage of participants with predominantly inattentive ADHD than the placebo group (7.7% vs. 20.4%). Mean (standard deviation [SD]) ADHD-RS-IV total and subscale scores were similar between DR/ER-MPH and placebo groups at baseline (total score: 43.4 vs. 41.6; inattention subscale: 22.3 vs. 21.9; hyperactivity/impulsivity subscale: 21.1 vs. 19.7). Therefore, despite categorical differences in CGI-S and ADHD presentation, the mean symptom severity, by total and subscale scores, was similar between the randomized groups.

**Table 1.** Demographic and Baseline Characteristics

	Enrolled safety population *N* = 125	Randomized safety population	
		DR/ER-MPH *n* = 65	Placebo *n* = 54
Gender, *n* (%)			
Male	85 (68.0)	42 (64.6)	38 (70.4)
Female	40 (32.0)	23 (35.4)	16 (29.6)
Age (years), mean (SD)	9.4 (1.65)	9.6 (1.58)	9.3 (1.68)
Age categories, *n* (%)			
6–7 Years	17 (13.6)	6 (9.2)	8 (14.8)
8–10 Years	73 (58.4)	38 (58.5)	32 (59.3)
11–12 Years	35 (28.0)	21 (32.3)	14 (25.9)
Race, *n* (%)			
White	99 (79.2)	50 (76.9)	45 (83.3)
Black/African American	15 (12.0)	9 (13.8)	5 (9.3)
Asian	0	0	0
Native Hawaiian/Pacific Islander	2 (1.6)	2 (3.1)	0
Other	9 (7.2)	4 (6.2)	4 (7.4)
Ethnicity, *n* (%)			
Hispanic/Latino	49 (39.2)	26 (40.0)	21 (38.9)
Non-Hispanic/Latino	76 (60.8)	39 (60.0)	33 (61.1)
Height (cm) at screening, mean (SD)	136.62 (11.426)	136.50 (10.651)	137.20 (12.414)
Weight (kg) at baseline, mean (SD)	32.71 (8.126)	33.04 (8.436)	32.65 (7.958)
ADHD presentation, *n* (%)			
Predominantly inattentive	17 (13.6)	5 (7.7)	11 (20.4)
Predominantly hyperactive-impulsive	0	0	0
Combined	108 (86.4)	60 (92.3)	43 (79.6)
CGI-S at baseline, *n* (%)			
Moderately ill (score of 4)	49 (39.2)	22 (33.8)	25 (46.3)
Markedly ill (score of 5)	53 (42.4)	26 (40.0)	24 (44.4)
Severely ill (score of 6)	22 (17.6)	16 (24.6)	5 (9.3)
Among the most extremely ill (score of 7)	1 (0.8)	1 (1.5)	0
ADHD-RS-IV (Total Score) at baseline, mean (SD)	42.7 (6.71)	43.4 (6.91)	41.6 (6.05)
CGI-P (Total Score) at baseline, mean (SD)	22.1 (5.25)	22.8 (5.28)	21.0 (4.82)

ADHD, attention-deficit/hyperactivity disorder; ADHD-RS-IV, ADHD Rating Scale Based on DSM-IV Criteria; CGI-S, Clinical Global Impressions-Severity; CGI-P, Conners' Global Index-Parent; DR/ER-MPH, delayed-release and extended-release methylphenidate; SD, standard deviation.

### DR/ER-MPH treatment optimization

After DR/ER-MPH treatment optimization, the mean (SD) daily optimized dose was 66.2 (19.56) mg, and the most common optimized administration time was 8:00 PM (64.1% of participants) in the ITT population. During the open-label, DR/ER-MPH treatment-optimization phase, 6 weeks of DR/ER-MPH treatment decreased mean (SD) ADHD-RS-IV from 42.5 (6.60) to 11.0 (7.14), BSFQ from 40.7 (10.28) to 7.3 (6.45), and CGI-P from 22.0 (5.11) to 5.5 (4.08). The mean percent reduction from baseline to Visit 8 was 74.0%, 82.2%, and 75.1% for ADHD-RS-IV, BSFQ, and CGI-P, respectively, demonstrating that most participants far exceeded the minimum improvement of 33% required for entry into the double-blind, placebo-controlled test phase. Additionally, the mean (SD) time to complete the early morning routine, from getting out of bed to exiting the home, decreased from 57.8 (30.39) minutes at baseline, following washout of previous ADHD treatment, to 42.7 (27.4) minutes at the end of the 6-week DR/ER-MPH treatment-optimization phase.

### Efficacy over the classroom day

**FIG. 3.**
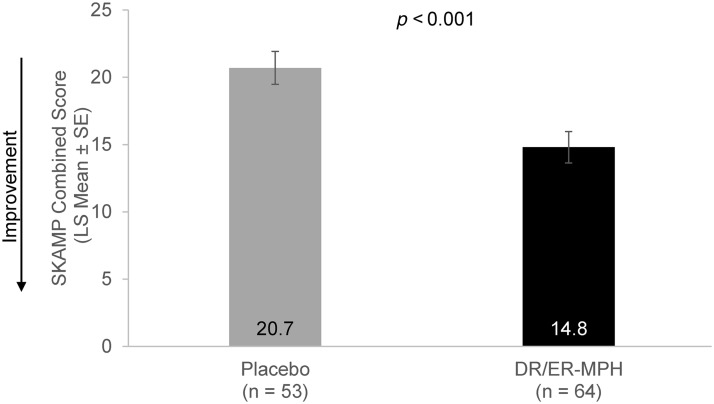
Model-adjusted average of all postdose SKAMP CS (LS Mean ± SE) from 8:00 AM to 8:00 PM at Visit 9. DR/ER-MPH, delayed-release and extended-release methylphenidate; LS, least squares; SE, standard error; SKAMP CS, Swanson, Kotkin, Agler, M-Flynn, and Pelham Scale combined score.

Following the 1-week double-blind phase, the study met its primary endpoint by showing a statistically significant improvement in the model-adjusted average of all postdose SKAMP CS from 8:00 AM through 8:00 PM in children treated with DR/ER-MPH versus placebo (LS mean [SE]: 14.8 [1.17] vs. 20.7 [1.22]; *p* < 0.001) (Fig. 3). The primary efficacy endpoint was also met for the sensitivity analysis population (the ITT population excluding Cohort 2 from Site B; DR/ER-MPH, *n* = 49; placebo, *n* = 51) (LS mean [SE]: 14.5 [1.28] for DR/ER-MPH vs. 21.0 [1.25] for placebo, *p* < 0.001).

**FIG. 4.**
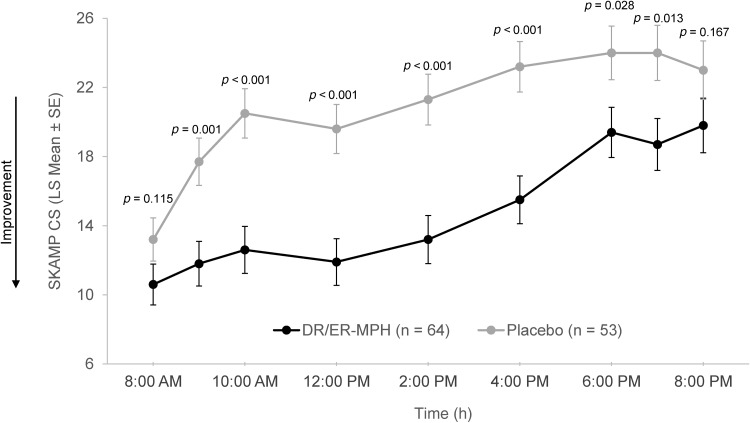
SKAMP CS from 8:00 AM to 8:00 PM at Visit 9. DR/ER-MPH, delayed-release and extended-release methylphenidate; LS, least squares; SE, standard error; SKAMP CS, Swanson, Kotkin, Agler, M-Flynn, and Pelham Scale combined score.

Treatment group differences in SKAMP CS scores were analyzed at every individual time point (8:00 AM, 9:00 AM, 10:00 AM, 12:00 PM, 2:00 PM, 4:00 PM, 6:00 PM, 7:00 PM, and 8:00 PM) over the laboratory classroom day (Fig. 4). Improvements in classroom behaviors for DR/ER-MPH versus placebo were statistically significant at the 9:00 AM (*p* = 0.001), 10:00 AM (*p* < 0.001), 12:00 PM (*p* < 0.001), 2:00 PM (*p* < 0.001), 4:00 PM (*p* < 0.001), 6:00 PM (*p* = 0.028), and 7:00 PM (*p* = 0.013) time points. At 8:00 AM and 8:00 PM, SKAMP CS favored children treated with DR/ER-MPH versus placebo; however, differences were not statistically significant (*p* = 0.115 and *p* = 0.167, respectively).

**FIG. 5.**
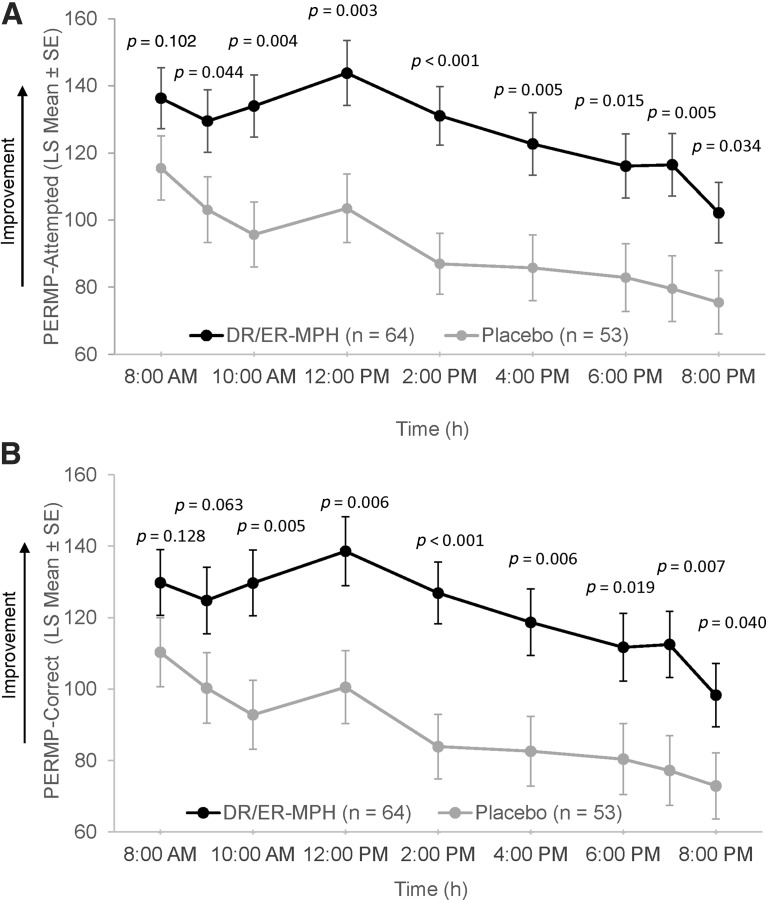
PERMP-attempted **(A)** and PERMP-complete **(B)** from 8:00 AM to 8:00 PM at Visit 9. DR/ER-MPH, delayed-release and extended-release methylphenidate; LS, least squares; PERMP, Permanent Product Measure of Performance; SE, standard error.

Improvements in behavior over the classroom day with DR/ER-MPH, as measured by SKAMP CS, were mirrored by improvements in effortful classroom performance, as measured by the number of problems attempted and correctly answered on the PERMP. Statistically significant improvements in model-adjusted average scores over all postdose time points were observed in children treated with DR/ER-MPH versus placebo on the PERMP-A (LS mean [SE]: 125.8 [8.78] vs. 92.1 [9.16]; *p* = 0.006) and PERMP-C (LS mean [SE]: 121.2 [8.78] for DR/ER-MPH vs. 89.0 [9.15]; *p* = 0.009). At individual time points, children treated with DR/ER-MPH showed statistically significant improvement in PERMP-A versus placebo (Fig. 5A) at 9:00 AM (*p* = 0.044), 10:00 AM (*p* = 0.004), 12:00 PM (*p* = 0.003), 2:00 PM (*p* < 0.001), 4:00 PM (*p* = 0.005), 6:00 PM (*p* = 0.015), 7:00 PM (*p* = 0.005), and 8:00 PM (*p* = 0.034); significant improvements with DR/ER-MPH versus placebo in PERMP-C (Fig. 5B) were observed at 10:00 AM (*p* = 0.005), 12:00 PM (*p* = 0.006), 2:00 PM (*p* < 0.001), 4:00 PM (*p* = 0.006), 6:00 PM (*p* = 0.019), 7:00 PM (*p* = 0.007), and 8:00 PM (*p* = 0.040).

### Efficacy during the early morning and late afternoon/evening

**FIG. 6.**
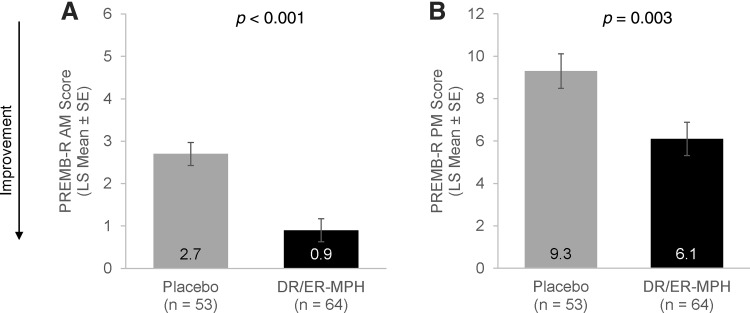
Early morning **(A)** and late afternoon/evening **(B)** functional impairment (LS mean ± SE) assessed at Visit 9. DR/ER-MPH, delayed-release and extended-release methylphenidate; LS, least squares; SE, standard error; PREMB-R AM, Parent Rating of Evening and Morning Behavior-Revised, Morning subscale; PREMB-R PM, Parent Rating of Evening and Morning Behavior-Revised, Evening subscale.

At Visit 9, after the 1-week double-blind phase, children treated with DR/ER-MPH versus placebo demonstrated improvements in EMF impairment, as measured by PREMB-R AM, the key secondary endpoint (LS mean [SE]: 0.9 [0.27] vs. 2.7 [0.27]; *p* < 0.001) (Fig. 6A). Significant reductions in late afternoon/evening functional impairment were also noted in the DR/ER-MPH group compared with the placebo group (LS mean [SE]: 6.1 [0.78] vs. 9.3 [0.81]; *p* = 0.003) (Fig. 6B). The sensitivity analysis of the ITT population, excluding Cohort 2 at Site B, also showed statistically significant improvements for DR/ER-MPH versus placebo in PREMB-R AM (LS mean [SE]: 0.8 [0.30] vs. 2.7 [0.29]; *p* < 0.001) and PREMB-R PM at Visit 9 (LS mean [SE]: 6.4 [0.89] vs. 9.4 [0.87]; *p* = 0.013).

### Safety

In the enrolled safety population (*N* = 125), the mean overall length of exposure to DR/ER-MPH was 44.0 days, with 40.4 days of exposure occurring during the open-label phase. For the 65 participants randomized to DR/ER-MPH, the mean exposure during the double-blind phase was 6.8 days. During the 6-week, open-label treatment-optimization phase, 111 participants (88.8%) experienced at least one TEAE (Table 2). The majority of the TEAEs were judged as mild or moderate in severity (maximum severity of any TEAE was mild in 50 participants [40.0%], moderate in 54 participants [43.2%], and severe in 7 participants [5.6%]). The severe TEAEs during the open-label, treatment-optimization phase were any insomnia; however, three of these participants had a prior medical history of insomnia, and all severe insomnia events resolved. No serious TEAEs were reported, and only three participants (2.4%) discontinued due to TEAEs (affect lability; anxiety/panic attack; agitation/aggression). The most common TEAEs (≥5%) during the open-label phase were any insomnia (40.8%), decreased appetite (27.2%), affect lability (21.6%), headache (17.6%), upper respiratory tract infection (16.0%), nausea or vomiting (8.8%), upper abdominal pain (8.8%), increased diastolic blood pressure (8.0%), tachycardia (7.2%), and irritability (6.4%).

**Table 2.** Treatment-Emergent Adverse Events Reported by ≥5% of Participants in Any Group and Vital Sign Changes from Baseline, Enrolled and Randomized Safety Populations

	Open-label phase	Double-blind phase	
n (%)	DR/ER-MPH N = 125	DR/ER-MPH n = 65	Placebo n = 54
Participants with at least one TEAE^a^	111 (88.8)	24 (36.9)	22 (40.7)
Any insomnia^b^	51 (40.8)	5 (7.7)	5 (9.3)
Decreased appetite	34 (27.2)	0	0
Affect lability	27 (21.6)	0	0
Headache	22 (17.6)	1 (1.5)	1 (1.9)
Upper respiratory tract infection	20 (16.0)	3 (4.6)	2 (3.7)
Nausea or vomiting	11 (8.8)	0	2 (3.7)
Upper abdominal pain	11 (8.8)	0	0
Increased diastolic blood pressure	10 (8.0)	9 (13.8)	7 (13.0)
Tachycardia	9 (7.2)	0	1 (1.9)
Irritability	8 (6.4)	0	0

Vital sign, mean (SD)	Change from baseline to the end of the open-label phase	Change from baseline to the end of the double-blind phase	
Systolic blood pressure, mmHg	6.2 (10.37)	6.3 (9.32)	3.6 (10.64)
Diastolic blood pressure, mmHg	6.5 (8.50)	5.1 (8.10)	5.9 (8.27)
Pulse, bpm	2.4 (13.55)	1.7 (11.12)	−0.2 (14.08)

^a^ Preferred terms are based on the Medical Dictionary for Regulatory Activities, Version 18.0 coding dictionary.

^b^ Includes the preferred terms insomnia (not specified), initial insomnia, middle insomnia, and terminal insomnia.

bpm, beats per minute; DR/ER-MPH, delayed-release and extended-release methylphenidate; SD, standard deviation; TEAE, treatment-emergent adverse event.

As expected, the incidence of TEAEs was lower during the 1-week, double-blind, placebo-controlled, DR/ER-MPH treatment-optimized test phase, with 24 participants (36.9%) in the DR/ER-MPH group and 22 participants (40.7%) in the placebo group experiencing at least one TEAE. No serious TEAEs were reported and no TEAEs led to discontinuation of the study drug. The most common TEAEs (≥5%) in DR/ER-MPH and placebo groups were increased DBP (13.8% vs. 13.0%) and any insomnia (7.7% vs. 9.3%).

Vital sign changes were consistent with those expected for MPH (Table 2). After 6 weeks of open-label DR/ER-MPH treatment, mean (SD) changes in the enrolled safety population from baseline to Visit 8 were 2.4 (13.55) beats per minute (bpm) for pulse, 6.2 (10.37) mmHg for systolic blood pressure, and 6.5 (8.50) mmHg for diastolic blood pressure. In the randomized safety population, mean (SD) changes in DR/ER-MPH versus placebo groups from baseline to Visit 9 were 1.7 (11.12) bpm versus −0.2 (14.08) bpm for pulse, 6.3 (9.32) mmHg versus 3.6 (10.64) mmHg for systolic blood pressure, and 5.1 (8.10) mmHg versus 5.9 (8.27) mmHg for diastolic blood pressure.

## Discussion

In the current study, significant improvements following 1 week of double-blind DR/ER-MPH treatment were reported versus placebo during the early morning, over a 12-hour laboratory school day, and during the late afternoon/evening, as assessed by the PREMB-R AM (key secondary endpoint), model-adjusted average of postdose SKAMP CS from 8:00 AM to 8:00 PM (primary endpoint), and PREMB-R PM (other secondary endpoint), respectively. Significant improvements with DR/ER-MPH versus placebo were reported in analyses of both the ITT and sensitivity populations (ITT population, excluding the 17 participants in Cohort 2 of Site B), suggesting that the randomization error of Cohort 2 at Site B did not affect outcomes. Thus, the inclusion of the 17 participants in the ITT, analyzed according to the treatment associated with the randomization number ultimately used, was appropriate. Consistent with improvements in classroom behavior, DR/ER-MPH treatment improved effortful performance, as indicated by average PERMP-A and PERMP-C scores over the classroom day versus placebo. Additionally, DR/ER-MPH was well tolerated, and the type of adverse events evidenced in the safety profile was consistent with other MPH formulations.

The study included three measures of functional impairment (PREMB-R AM, SKAMP, and PREMB-R PM) to support efficacy of DR/ER-MPH across the waking day. These measures are validated to capture distinct activities across settings and reflect a child's daily experience on different days. Furthermore, these scales assess overlapping temporal periods: PREMB-R AM evaluates the mornings (with items that include “Getting out of bed”), SKAMP evaluates the time from 8:00 AM to 8:00 PM, and PREMB-R PM evaluates the late afternoon/evening (with items that include “Settling down/getting ready for bed” and “Falling asleep”). The questions in these scales do not form a single ecologically valid instrument relying on time sampling; therefore, the duration of effect for DR/ER-MPH can only be approximated from the general temporal periods over which the scales are rated—from waking to the evening.

The time to complete the morning routine, measured from the BSFQ, was assessed at baseline and the end of the 6-week open-label period to understand additional aspects of the morning differences between children in untreated and treated states. The mean (SD) time to complete the early morning routine was 57.8 (30.39) minutes at baseline (i.e., following washout of previous ADHD treatment) and 42.7 (27.40) minutes at the end of the 6-week DR/ER-MPH treatment-optimization phase. Although the significance, if any, of these changes are unknown, and further exploration is warranted, these data highlight the short (<1 hour) timeframe in which children with ADHD must complete activities of daily living before leaving the house. Because an early morning routine of at least 30 minutes was an inclusion criterion of the study, the time from waking to leaving the house may be even shorter in the wider population of children with ADHD. Based on the short time window reported in this study, control of ADHD during the early morning routine remains a significant unmet need in MPH-treated youth with ADHD.

Although the trial met its primary endpoint of model-adjusted average of postdose SKAMP CS from 8:00 AM to 8:00 PM, when SKAMP CS was assessed at each individual time point over the classroom day (secondary endpoint), mean SKAMP CS at 8:00 AM and 8:00 PM were lower for the DR/ER-MPH group versus the placebo group but not statistically different. Despite not reaching statistical significance compared with placebo, the greatest control of classroom behaviors in the DR/ER-MPH group was seen at the 8:00 AM SKAMP CS time point and was consistent with the benefit seen at 9:00 AM (LS mean [SE] of 10.8 [1.20] and 11.9 [1.13], respectively) (Fig. 4), suggesting that the lack of significance at the earliest morning time point was not driven by lack of DR/ER-MPH treatment effect per se but by relatively good control of behaviors reported in the placebo group, due possibly in part to a higher number of predominantly inattentive presentation participants and lower clinical severity based on CGI-S scores, as described earlier. Moreover, the laboratory classroom is a tightly controlled environment, where subjects are closely monitored with redirections and prompts in contrast to the morning routine at home where children are expected to complete tasks (e.g., getting dressed, eating breakfast, brushing teeth) either unsupervised or without constant supervision and have more opportunity for distraction (e.g., toys, television, video games, phone).

Although the SKAMP time course for the placebo group looked similar to those of other MPH studies, the DR/ER-MPH time course demonstrated that classroom behaviors measured with the SKAMP were controlled at the earliest time point with DR/ER-MPH (Fig. 4). In time course effects on the SKAMP observed with other MPH formulations (Wigal et al. 2013, 2017; Neos Therapeutics 2017; Rhodes Pharmaceuticals 2017; Purdue Pharmaceuticals 2019), worse behavior has been observed in the MPH-treated group compared with the placebo-treated group at the first morning measurement ( predose), an effect not evidenced in this study due to evening dosing. However, analyses across studies should be interpreted with caution given that there are no head-to-head studies comparing the clinical safety and efficacy of DR/ER-MPH and other MPH products.

As functional impairment manifests across different settings—at home, school, work, or extracurricular/leisure activities—the optimal evaluation of functional impairment includes a multidimensional approach across multiple settings with multiple informants (e.g., parents, teachers) to complement reports and minimize bias (Barkley 2014; Sasser et al. 2017). In the current trial, the importance of using complementary measures became apparent when interpreting outcomes during the early morning and late afternoon/evening. Even though the mean SKAMP CS were not significantly improved in the DR/ER-MPH group versus placebo when analyzed at the 8:00 AM and 8:00 PM time points, significant improvements with DR/ER-MPH versus placebo on the PREMB-R (both AM and PM subscales) were consistent with a treatment effect covering the early morning and lasting into the evening, at least when rated on different days of the study in a nonclassroom setting by investigators based on parent interviews regarding at-home and extracurricular activities.

Results from this trial showed DR/ER-MPH to be well tolerated at doses ranging from 20 to 100 mg/day. As expected, and consistent with other MPH trials with a dose-optimization phase (Wigal et al. 2013, 2014, 2017; Childress et al. 2017), a higher incidence of TEAEs occurred during the open-label, DR/ER-MPH treatment-optimization phase versus the double-blind phase (Table 2). This trend is partly due to the standardized method in which TEAEs are recorded in trials with an open-label phase followed by a double-blind phase: a TEAE occurring in the open-label phase and continuing without worsening into the double-blind phase is recorded only in the open-label phase. Additional factors influencing higher open-label TEAE rates include typically longer duration and the process of dose optimization per se aiming to ensure that the most effective and tolerable dose is selected for the double-blind phase. After DR/ER-MPH treatment optimization, TEAEs during the double-blind phase were mild/moderate and balanced between DR/ER-MPH and placebo groups; no serious TEAEs or TEAEs leading to premature withdrawal were reported. During both study phases, the most frequently reported TEAEs were generally consistent with those typically reported with MPH treatment (Table 2).

Sleep-related TEAEs were prespecified as TEAEs of special interest, and at each visit, onset, quality, and quantity of sleep were directly queried from participants/caregivers. The frequency of any type of insomnia during the 6-week, open-label, treatment-optimization phase was 40.8%; however, none of the open-label insomnia events led to discontinuation. During the double-blind phase, the proportion of participants reporting any insomnia was similar between groups (7.7% in the DR/ER-MPH group and 9.3% in the placebo group). Higher absolute rates of sleep-related TEAEs in both DR/ER-MPH and placebo groups were expected in this study given that sleep disturbances were directly queried at each visit, compared with similarly designed MPH studies in which sleep-related TEAEs were collected using spontaneous report, a trend that has been previously described (Wernicke et al. 2005).

As described in a recent meta-analysis of sleep-related TEAEs reported in double-blind MPH trials in naturalistic settings, the appropriate and meaningful comparison of insomnia across studies requires the comparison of relative risk (RR; ratio of the probability of insomnia in the treated group to the probability of insomnia in the placebo group) adjusted for the absolute rate of insomnia in the placebo arm and various study design and sample features (Faraone et al. 2019). One of the features that confounds RR is percentage of stimulant responders, as studies that enroll a large fraction of stimulant responders have lower RRs than other studies due to the definition of “response” including tolerability. The authors also conclude that without a placebo comparator, it is not possible to appropriately compare absolute open-label rates of insomnia between trials. In the recent meta-analysis, after taking into account confounding study design and sample features as well as the placebo sleep-related adverse event rate, the model-adjusted RR of sleep-related adverse events with DR/ER-MPH from the pivotal, phase 3, forced-dose titration study (Pliszka et al. 2017) was in line with other MPH formulations as reported in double-blind trials in naturalistic settings (Faraone et al. 2019).

The results of this trial are consistent with the other pivotal, phase 3, randomized, double-blind, placebo-controlled, forced-dose titration study in a naturalistic setting in children 6–12 years of age with ADHD (Pliszka et al., 2017). In that study, 3 weeks of DR/ER-MPH treatment was well tolerated and resulted in significant improvements in ADHD-related symptoms versus placebo, as measured by ADHD-RS-IV. Additionally, DR/ER-MPH significantly improved EMF impairment, as measured by two instruments, the PREMB-R AM and the BSFQ (Wilens et al. 2010; Faraone et al. 2018b). As in the current study, significant improvements in late afternoon/evening functional impairment were reported with DR/ER-MPH based on PREMB-R PM. Therefore, improvements in early morning and late afternoon/evening functional impairment with DR/ER-MPH versus placebo have now been replicated in two pivotal trials of DR/ER-MPH with varying study designs and by three validated instruments—two measuring functional impairment during the early morning (BSFQ and PREMB-R AM) and one measuring functional impairment during the late afternoon/evening (PREMB-R PM).

The results of this study should be considered in light of several study design limitations. First, the exclusion of a study site (*n* = 36) may have reduced power to detect significant differences at some secondary endpoints. Furthermore, differences in baseline characteristics, with more children diagnosed with predominantly inattentive only and with less clinical severity based on the CGI-S in the placebo group, may have also affected treatment differences. Moreover, the study included only school-age children (6–12 years of age) with a history of at least partial response to MPH and most psychiatric comorbidities were excluded. Therefore, the applicability of these findings to other age groups (i.e., preschool children, adolescents, and adults), MPH-naive, and other presentations of ADHD is unknown. Also, this laboratory school protocol study, as is typical, includes only children with ADHD, unlike a typical classroom, and is more structured and lengthier, which allows for extended observations compared with a typical elementary school classroom. Additionally, the study was designed to assess functional impairment across settings using multiple measures which capture overlapping periods across the day, although collected on different days, starting from the time of waking; therefore, duration of effect can only be inferred from the general temporal periods over which the measures of functional impairment were assessed. Finally, the study was not designed to assess long-term safety or efficacy.

## Conclusions

Evening-dosed, treatment-optimized DR/ER-MPH significantly improved ADHD-related functional impairment in the before-school early morning period, during the laboratory classroom period, and in the late afternoon/early evening period compared with placebo in children with ADHD. Treatment-optimized DR/ER-MPH was well tolerated: during the double-blind phase there were no discontinuations due to TEAEs, TEAEs did not differ between DR/ER-MPH and placebo groups, and all TEAEs were mild/moderate.

## Clinical Significance

Evening-dosed DR/ER-MPH represents a shift in the approach to the timing of MPH delivery to target efficacy upon awakening, throughout the day, and into the evening. While other formulations have sought to reduce the interval between oral dosing and onset of effect, morning-dosed MPH formulations may still have an inherent lag before therapeutic effect (Sallee 2015; Childress 2016; Faraone et al. 2017). Because there is no single ecologically valid measure to capture ADHD manifestations throughout the day, the use of multiple measures of functional impairment across the day may be, at present, the most appropriate way to estimate duration of treatment effect throughout the entire waking day—both in clinical trials and actual practice.
